# Cutaneous Allodynia in Migraine: A Narrative Review

**DOI:** 10.3389/fneur.2021.831035

**Published:** 2022-01-21

**Authors:** Ane Mínguez-Olaondo, Sonia Quintas, Noemí Morollón Sánchez-Mateos, Alba López-Bravo, Marta Vila-Pueyo, Vesselina Grozeva, Robert Belvís, Sonia Santos-Lasaosa, Pablo Irimia

**Affiliations:** ^1^Neurology Department, Hospital Universitario Donostia, San Sebastián, Spain; ^2^Athenea Neuroclinics, Policlínica Guipúzcoa, Grupo Quirón Salud Donostia, San Sebastián, Spain; ^3^Neuroscience Area, Biodonostia Health Institute, Donostia, Spain; ^4^Medicine Faculty, University of Deusto, Bilbao, Spain; ^5^Clínica Universidad de Navarra, Pamplona, Spain; ^6^Hospital Universitario de la Princesa, Madrid, Spain; ^7^Headache and Neuralgia Unit, Neurology Department, Hospital de la Santa Creu i Sant Pau, Barcelona, Spain; ^8^Hospital Reina Sofía, Tudela, Spain; ^9^Instituto de Investigación Sanitaria Aragón, Zaragoza, Spain; ^10^Headache and Neurological Pain Research Group, Department of Medicine, Vall d'Hebron Research Institute, Universitat Autònoma de Barcelona, Barcelona, Spain; ^11^Private Neurology Practice, Sofia, Bulgaria; ^12^Hospital Clínico Universitario Lozano Blesa, Zaragoza, Spain

**Keywords:** cutaneous allodynia, migraine, migraine chronification, risk factors, treatment

## Abstract

**Objective:**

In the present work, we conduct a narrative review of the most relevant literature on cutaneous allodynia (CA) in migraine.

**Background:**

CA is regarded as the perception of pain in response to non-noxious skin stimulation. The number of research studies relating to CA and migraine has increased strikingly over the last few decades. Therefore, the clinician treating migraine patients must recognize this common symptom and have up-to-date knowledge of its importance from the pathophysiological, diagnostic, prognostic and therapeutic point of view.

**Methods:**

We performed a comprehensive narrative review to analyze existing literature regarding CA in migraine, with a special focus on epidemiology, pathophysiology, assessment methods, risk for chronification, diagnosis and management. PubMed and the Cochrane databases were used for the literature search.

**Results:**

The prevalence of CA in patients with migraine is approximately 60%. The mechanisms underlying CA in migraine are not completely clarified but include a sensitization phenomenon at different levels of the trigemino-talamo-cortical nociceptive pathway and dysfunction of brainstem and cortical areas that modulate thalamocortical inputs. The gold standard for the assessment of CA is quantitative sensory testing (QST), but the validated Allodynia 12-item questionnaire is preferred in clinical setting. The presence of CA is associated with an increased risk of migraine chronification and has therapeutic implications.

**Conclusions:**

CA is a marker of central sensitization in patients with migraine that has been associated with an increased risk of chronification and may influence therapeutic decisions.

## Introduction

Cutaneous allodynia (CA) is defined as pain resulting from application of a non-noxious stimulus to normal skin (1, 2), and is classified according to either the sensory modality that is used to elicit the sensation (thermal, mechanical dynamic, and mechanical static allodynia) or by the location of the symptom (cephalic and extracephalic). The presence of CA in patients with migraine was described more than 100 years ago (3), but only recently a growing number of studies began to highlight the importance of this common and bothersome symptom (2, 4–6). Therefore, the clinician treating migraine patients must recognize CA (7) and have up-to-date knowledge of CA importance from pathophysiological, diagnostic, prognostic and therapeutic point of view.

In the present work, we performed a comprehensive narrative review to analyze the most relevant literature on CA in migraine, with a special focus on epidemiology, pathophysiology, assessment methods, risk for chronification, diagnosis, and management.

## Methods

We conducted a non-systematic literature search in PubMed and Cochrane databases for published manuscripts on allodynia and migraine in October 2021, with no date limits. Search terms were limited to migraine and allodynia. The articles were selected independently by the authors based on the intrinsic characteristics and value of the studies. Articles addressing epidemiology, pathophysiology, evaluation tools, risk for chronification, diagnosis and management in patients with CA were included. The search included publications in English and Spanish.

## Epidemiology of Allodynia in Migraine

The prevalence of CA in migraine is highly variable (8, 43) in the literature (Table 1), although it can be considered that between 40 and 70% of the patients with migraine experience this symptom, according to population-based studies (26, 36, 37). Several factors influence these differences including the heterogeneity of the populations in each study (population-based nationwide studies, cross-sectional studies or prospective case series) and the methodology used for the identification of CA (8–43).

**Table 1:** Most relevant studies regarding different epidemiological aspects of CA in migraine.

**References**	**Year**	**Number of patients**	**Country**	**Type of study**	**Type of migraine**	**Medication overuse**	**Age range**	**% of patients with CA**	**Evaluation method**
Han et al. (8)	2021	289	Korea	Cross-sectional study	PM	Not specified	>18 years	17%	ASC-12
Akarsu et al. (9)	2019	871	Turkey	Nationwide population-based study	Migraine	Not specified	>17 years	57.5%	Not described
Munjal et al. (10)	2019	6,045	U.S.	Cross-sectional observational	Migraine	Not specified	18–65 years	61.1%	ASC-12
Lipton et al. (11)	2019	8,658	U.S.	Longitudinal, internet-based panel	EM	Included, 19%	>17 years	36.7%	ASC-12
Young et al. (12)	2019	716	U.S., Australia and South Korea	Multileft, open-label, prospective study	CM	Icluded, 11.9%	Not described	44.4%	ASC-12
Seo et al. (13)	2019	312	South Korea	Cross-sectional study	Migraine	Independently assessed, 20.7% Vs 11.9% in those with allodynia	Not described	39.9%	ASC-12
Lipton et al. (14)	2018	18,353	U.S.	Longitudinal, internet-based panel	Migraine	Not specified	>17 years	40.4%	ASC-12
Lipton et al. (15)	2018	12,810	U.S.	Longitudinal, internet-based panel	CM and EM	Included	15–70 years	18.6%	ASC-12
Levinstky et al. (16)	2019	119	Israel	Retrospective transversal study	Migraine 3 – 18 years	Included, 2.8%	>17 years	39.9%	ASC-12
De Tommasso et al. (17)	2017	151	Italy	Observational cross-sectional study	CM, EM 8 - 15years	Included, 15%	Not described	47%	ASC-12
Kim et al. (18)	2016	140	South Korea	Prospective consecutive patients	CM, EM	Not specified	3–18 years	31.1%	ASC-12
Mathew et al. (19)	2016	44	US	Consecutive patients	CM	Not specified	8–15 years	96.6%	ASC-12
Mendonça et al. (20)	2016	98	Portugal	Cross - sectional study	EM	Included, 19.5%	15–75 years	14.5%	ASC-12
Baykan et al. (21)	2016	871	Turkey	Population-based nationwide, home-based prevalence study in adults	Migraine	Not specified	Not described	91%	Standard questions
Lovati et al. (22)	2015	673	Milan, Italy	Prospective consecutive patients	CM and EM	Not specified	Not described	77%	Standard questions
Park et al. (23)	2015	220	Korea	Cross-sectional study	CM, EM and MOH	Independently assessed, higher in those with allodynia (10.3% Vs 4.7%)	Not described	61.1%	ASC-12
Raieli et al. (24)	2015	202	Italy	Retrospective study	Migraine Children and teenagers	Not specified	Not described	50%	Brief questionnaire
Baldacci et al. (25)	2015	200	Italy	Cross-sectional study	Migraine	Included, 23.6%	15 - 73 years	47.3%	ASC-12
Louter et al. (26)	2013	2,331	The Netherlands	Population-based nationwide survey	Migraine	Not specified	Children and teenagers	37.1%	ASC-12
Kao et al. (27)	2014	434	Taiwan	Cross-sectional study	Migraine	Excluded	>17 years	79%	ASC-12
Menon et al. (28)	2013	422	India	Cross-sectional study in medical students	Migraine	Included	18–74 years	70%	Not described
Lovati et al. (29)	2013	456	Italy	Prospective consecutive patients	CM and EM	Not specified	Not described	48.4%	Semi-structured ad hoc questionnaire
Misra et al. (30)	2013	448	India	Prospective consecutive patients	Migraine	Included, 27%	Not described	23%	Own check list
Güven et al. (31)	2013	168	Turkey	Prospective consecutive patients	EM	Excluded	Not described	54.4%	ASC-12
Díaz-Insa et al. (32)	2011	1,036	Europe	Randomized placebo-controlled trial	Migraine	Not specified	10–62 years	71.4%	Not described
Chou et al. (33)	2010	100	Taiwan	Cross-sectional study	CM and EM	Not specified	>18 years	61.3%	Self-reported brushing allodynia
Tietjen et al. (34)	2009	1,413	U.S.	Cross-sectional study	Migraine	Not specified	Not described	39%	Questionnaire
Friedmann et al. (35)	2009	182	U.S.	Multileft ED-based clinical trial	Migraine in the emergency department	Not specified	17–65 years	17%	BA
Bigal et al. (36)	2008	11,094	U.S.	Population-based nationwide survey AMPP	TM and EM	Included, 6%	>17 years	60%	ASC-12
Lipton et al. (37)	2008	11,388	U.S.	Population-based nationwide study	Migraine	Not specified	Not described	14%	ASC-12
Lovati et al. (38)	2007	221	Italy	Consecutive patients	EM and CM	Not specified	>17 years	68.3% and 63.25	Not described
Ashkenazi et al. (39)	2007	151	U.S.	Cross-sectional study	EM	Not specified	>17 years	63%	QST
Ashkenazi et al. (40)	2007	89	U.S.	Cross-sectional study	CM	Included	Not described	>40%	BA
LoPinto et al. (41)	2006	55	U.S.	Cross-sectional study	Migraine	Not specified	12–60 years	77%	Cutaneous stimulation (BA) And pressure alllodynia (PA)
Raieli et al. (42)	2005	55	Italy	Cross-sectional study	Migraine 6 - 18 years	Not specified	>15 years	42.7%	Not described
Mathew et al. (43)	2004	295	U.S.	Cross-sectional study	Migraine	Not specified	Not described	32.7%	Semi-structured ad hoc questionnaire
								14.5%	
								53.3%	

*ASC-12, allodynia symptom checklist; BA, brushing allodynia; CM, chronic migraine; EM, episodic migraine; PM, probable migraine (fulfills all but one criterion of migraine); QST, quantitative sensory test; TM, transformed migraine*.

Two of the largest population-based studies were conducted in the United States to assess the prevalence of CA in patients with migraine (36, 37). Prevalence rates range from 39 to 63% (36, 37). In both studies, migraine diagnosis was made using validated questionnaires, not through a medical consultation, and 12-item Allodynia Symptom Checklist (ASC-12) was used to detect CA. The population studies published to date have large sample sizes that allow the analysis of patients by subgroups and facilitate the study of the influence of different clinical and sociodemographic variables on the appearance of CA. In this sense, a higher prevalence of CA has been observed in women, in patients with frequent migraine and in those with anxiety and/or depression, and acute medication overuse (36, 37, 44). Some studies also found that CA increased with body mass index (BMI) (34, 45, 46). Furthermore, Louter et al. (26) developed a study with a similar design in the Netherlands and found a 70% prevalence of CA in patients with migraine. The risk factors identified for developing CA, included lifetime depression, female gender, low age at onset, and high migraine attack frequency, but not BMI. Other investigators have studied the prevalence of migraine in cohorts of patients recruited from a single-center and with a limited number of subjects (Table 1), which makes it difficult to generalize the results and explain, at least in part, the wide range found in the prevalence of CA.

One of the findings that is consistently observed in most studies is that CA increased with headache frequency suggesting that repeated migraine attacks lead to the development of allodynia. Some studies also describe that the presence of AC is more frequent in patients with migraine with aura (37) and in those who overuse analgesics (44).

In the coming years, the widespread use of the ASC-12 scale will allow us to have more data on the prevalence of CA. It would be very useful to have data obtained in large series of patients, attended in different centers, in which the diagnosis of migraine has been made by a specialist in a face-to-face interview.

## Pathophysiology of Allodynia in Migraine

**Figure 1:**
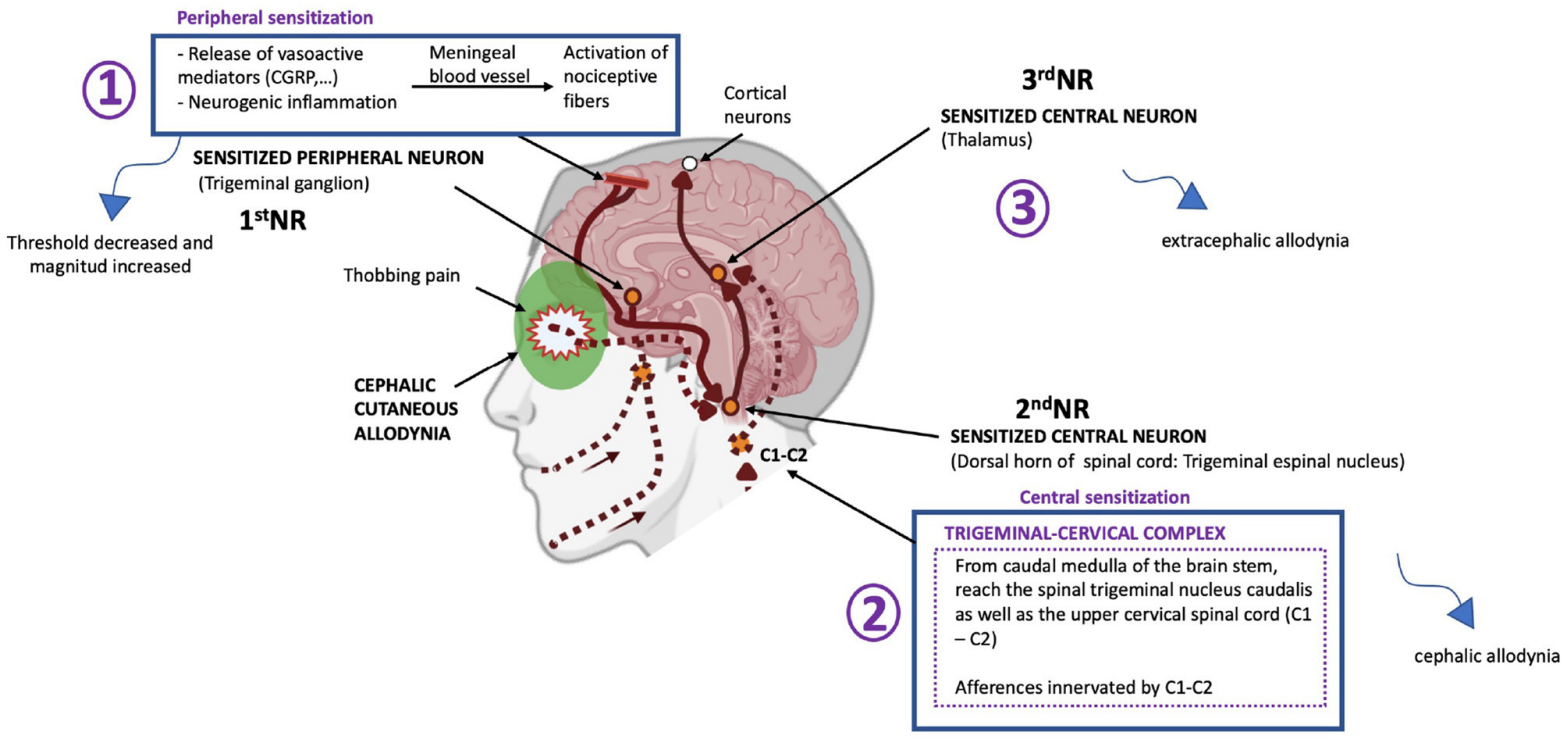
Schematic representation of the sensitization of central trigeminovascular neurons in the thalamus believed to mediate the extracephalic CA trigeminal and nucleus caudalis believed to mediate cephalic CA during migraine. NR, Neuron. Adapted from Noseda and Burstein (72). Biorender.com

The mechanisms underlying CA in migraine are still not completely understood but include a sensitization phenomenon at different levels of the trigemino-vascular system and ascending projections, and a dysfunction of the different brainstem and cortical areas that modulate thalamocortical inputs (47, 48) (Figure 1). The trigeminal ganglion (TG) gives rise to pseudo-unipolar trigeminal primary afferents that project to peripheral and central sites. In the periphery, the nociceptive fibers from TG neurons innervate the meninges, dural blood vessels, middle meningeal artery, and large cerebral arteries. Activation and sensitization of these peripheral structures occurs by the local release of vasoactive peptides, such as calcitonin gene-related peptide (CGRP), amylin, substance P and pituitary adenylate cyclase-activating peptide (PACAP) (49–52), and inflammatory mediators (49). Sensitization of peripheral afferent TG fibers, or peripheral sensitization, explains the characteristic throbbing pain that worsens with physical activity during migraine attacks (49). Peripheral sensitization also implies that the threshold needed by these first-order neurons of the TG to respond to a stimulus decreases and that the magnitude of the response is increased (47).

There are central afferent projections from the TG neurons to the trigeminal nucleus caudalis (TNC) and the upper cervical spinal cord (C1–C2) (53). The TNC receives repetitive, nociceptive inputs from the periphery and become sensitized to further stimulus. Central sensitization refers to a condition in which central neurons in the trigeminal nociceptive pathway, mainly TNC second-order neurons or third-order neurons in the thalamus, are sensitized and exhibit increased excitability (2, 43). Sensitization of second-order neurons of the TNC explain the appearance of cephalic allodynia in patients with migraine (47, 49). It has been observed that once the sensitization of second order trigeminovascular neurons is established, it becomes activity independent, and it maintains itself in the absence of sensory input (54).

Subsequently, nociceptive information ascends from the TNC toward the thalamus. Sensitization of third-order thalamic neurons (especially those located in the pulvinar region) is the mechanism that explains extracephalic allodynia (2, 47, 49).

Additionally, functional MRI studies also show that the activation pattern of cortical structures after a painful stimulus is different in patients with migraine and allodynia. These findings support the existence of a dysfunction of the trigeminal-thalamus-cortical nociceptive pathway, which could specifically affect the cortical areas that modulate thalamocortical inputs (48, 55).

Apart from noxious stimuli, sensitization can be activated other mechanisms. Experimentally, it has been observed that CSD is capable of activating peripheral and central trigeminovascular neurons (56). Also, fat excess, may promote chronic release of different inflammatory mediators that decrease the threshold for the onset of a migraine attack and contribute to central sensitization (46). Finally, central sensitization may also be facilitated by the impairment of descending inhibitory pain mechanisms on the trigeminovascular system observed in patients with migraine (57).

## Assessment of Allodynia in Migraine Patients

CA is usually classified according to the type of stimulus that causes the sensation of pain. At least three types of CA have been consistently described: thermal allodynia, dynamic mechanical, and static mechanical (1, 7). Thermal allodynia is mediated by nociceptive fibers C and Aδ (1, 7) and is usually assessed with Quantitative Sensory Testing (QST). Dynamic mechanical allodynia is mediated by Aβ mechanoreceptive and Aβ fibers insensitive to capsaicin (7) and is assessed by brushing the skin. Finally, static mechanical allodynia is mediated by Aδ nociceptive fibers (58) and evaluated using von Frey hair filaments.

QST is the gold standard method for assessing allodynia. However, this neurophysiological test requires special equipment, is time-consuming and costly for use in clinical settings. Therefore, initial studies on CA were conducted in very few centers and included highly selected patients (2, 59, 60). In order to have a method for measuring CA in clinical practice, Lipton et al. developed and validated a questionnaire for assessing CA. The Allodynia Symptom Checklist or ASC-12 (37), was developed by modifying Jakubowski et al. instrument (7) to provide graded allodynia response options, as CA is not an all or none phenomenon. Based on clinical interview, different types of allodynic symptoms could be studied during or immediately after the migraine attacks including cephalic (scalp, face, neck, ears) and extracephalic symptoms (limbs, trunk) (43). The aim was to quantify CA in general based on clinical symptoms and determine if there were natural subtypes of CA (37). ASC-12 has high reliability in detecting patients with CA but low reliability in detecting patients without CA (7). Perhaps the addition of questions aimed at improving this aspect could give the tool even greater value. Nevertheless, this questionnaire helps neurologists identify patients with CA within the time frame of a routine office visit even when the patient is free of migraine (7).

The ASC-12 includes 12 questions on the frequency of various symptoms of CA in association with a headache attack. For people with more than one type of headache, questions are focused on the “most severe type of headache,” as it is likely to be migraine (61). The response categories are “never,” “rarely,” “less than half the time,” and “half the time or more.” According to previous studies, the option “rarely” is considered a negative answer and the objective of its presence is to reduce false positives (37, 62, 63). Additionally, subjects can also indicate that an item “does not apply to me” that option is for someone who has never shaved their face or someone who has never worn a ponytail. The ASC-12 items are scored as 0 (that is, never, rarely, or do not apply to me), 1 (less than half the time), and 2 (half the time or more), so the score can range from 0 to 24 and in its validation the following categories are defined based on the total scores obtained, being: non-allodynia 0–2, mild allodynia 3–5, moderate allodynia 6–8 and severe allodynia ≥ 9 (37).

## Use of Allodynia as a Readout of Migraine-like Pain in Preclinical Models

Preclinical models have greatly enhanced our current understanding of migraine; hence they are considered an invaluable tool for investigating this disorder. One of the most used and easiest to quantify readout in preclinical models of migraine is CA (64), which indicates the importance attributed to CA within the field of migraine research. CA can be measured in preclinical models by using either mechanical or thermal sensory nociceptive thresholds, which have been shown to be reliable markers of migraine pathophysiology (65).

Mechanical allodynia is the most used method for studying pain-like behavior in preclinical models of headache, and it is assessed with the von Frey test. This method uses calibrated von Frey filaments which are applied to the periorbital region (cephalic) or hind paw (extracephalic) of rodents to determine evoked response thresholds (64). Von Frey test is used to assess responses to different substances, such as the dural inflammatory soup and the administration of algogenic substances, nytroglicerin, CGRP, amylin or pituitary adenylate cyclase-activating polypeptide (PACAP), among others (66, 67). The translational utility of this test has been crucial to investigate the underlying mechanisms of several migraine treatments and to predict the efficacy of new therapies. Some of the treatments positively tested to date include triptans (68, 69), which in turn are also used as a positive control in these tests; gepants (69); CGRP monoclonal antibodies (69); ditans (70); and opioid receptor agonists (71).

Although most preclinical studies are focused on assessing mechanical allodynia, thermal allodynia (cold and hot) can also be easily assessed, and both measures can complement each other to enhance our understanding of the mechanisms involved in migraine pathophysiology. Thermal allodynia can be measured with different methodologies including the Hargreaves' test (73), based on applying a heat stimulus to the hind paws or tail of the animal to measure their withdrawal latency; and the acetone evaporation test, which measures the aversive behaviors elicited by evaporative cooling both in cephalic or extracephalic regions (74). These tests have been used in preclinical models of migraine, where the administration of nitroglycerin and algogenic substances have shown to increase thermal allodynia (67, 68), and the nasocilary nerve ligation to increase sensitization to acetone evaporation (75).

Basic science has proven to be a fundamental pillar for the development of knowledge in migraine and in the relevance of CA in it. Hence the value of continuing to use animal models that resemble the human and thus be able to deepen the knowledge on this area.

## Allodynia and Migraine Chronification

The presence of CA is currently considered as a possible marker and a risk factor for migraine chronification (19, 26) (Table 2). CA is more common in patients with frequent attacks, and long-lasting disease (19, 26, 34, 36, 37). The increased headache frequency, disability, and longer duration of illness indicate that either CA is a marker for a more severe migraine disorder or that repeated attacks over a long period can lead to development of CA (26, 34).

**Table 2:** Summary of the studies analyzing the prevalence of CA in Episodic Migraine (EM) vs. Chronic Migraine (CM) (in %) and those identifying CA as a risk factor for migraine chronification (NA, Not Applicable; TTH, Tension Type Headache; HTTH, Chronic Tension Type Headache).

**References**	**Year**	**Number of patients**	**Country**	**CA as a risk factor for chronification**	**Type of migraine**	**CA prevalence in EM %**	**CA prevalence in CM %**
Russo et al. (55)	2020	50	Italy	YES	Migraine	NA	NA
Mathew et al. (19)	2016	44	U.S.	YES	CM	–	~90%
Louter et al. (26)	2013	3,029	Netherlands	YES	Migraine	–	–
Tietjen et al. (34)	2009	1,413	U.S.	NA	Migraine	–	–

The association between CA and migraine frequency may be explained by the repetitive activation of trigemino-talamo-cortical nociceptive pathway. Recurrent migraine attacks with CA leads to a decreased threshold for subsequent migraine attacks (76) but may also induce functional changes in different structures involved in pain modulation, such as periaqueductal gray (26, 36), the spinal trigeminal nucleus and posterior thalamus (77). Rome et al. give another explanation using a nociception-induced plasticity model suggesting that a noxious stimulus may, under certain conditions, lead to a sensitized corticolimbic state, and pain chronification (26).

On the other hand, lower thermal pain thresholds on cutaneous testing have been noted during an attack among patients with migraine and CA (19). When tested with a functional magnetic resonance imaging (MRI) study using blood oxygen-level-dependent (BOLD) contrast-sensitive sequences, the patients with migraine with CA showed more prominent periaqueductal gray and nucleus cuneiformis resting state connectivity with other brainstem, thalamic, insula, and cerebellar regions that process pain when compared to patients with migraine with no-CA (19). Resting state connectivity in this population was also found to be stronger, with frontal and temporal regions suggesting differences in higher-order pain modulation (78). These thermal threshold and MRI BOLD imaging data further clarify that the presence of CA is probably a risk factor for the transformation from episodic to chronicmigraine (19). A deeper understanding of CA and how it is related to chronification of migraine may elucidate the exact pathophysiological mechanisms and can provide a rationale for adequate treatment of this difficult to manage migraine form (76).

## Implications of Allodynia for Migraine Treatment

The efficacy of the treatment of migraine attacks is reduced in patients with CA (79). Data from the AMPP study observed that CA has been associated with an increased likelihood of an inadequate response to triptans, non-steroidal anti-inflammatory drugs, opioids, and barbiturates in comparison with patients without CA (80). However, randomized controlled trials did not confirm these findings (81, 82). The impact of CA on treatment outcomes in the patients with acute migraine treated with almotriptan was analyzed in the “Act when Mild” study. The presence of CA did not alter the efficacy of almotriptan administered for early/mild pain in terms of 2-h pain-free rates or sustained pain-free rates. CA impaired the effectiveness of almotriptan in patients with moderate to severe pain in terms of longer migraine duration. Therefore, the data from this study suggest that pain intensity is the main driver of triptan response, and not the presence of CA (32).

Available data are not finally conclusive regarding the role of CA in triptan response (81, 82). A fundamental question may be the distinction between developing CA, in which central sensitization depends on incoming pain signals from peripheral nociceptors, and established CA, where sensitization becomes independent of pain signals coming from the dura mater (83).

The influence of CA on the response to preventive treatment is currently unknown. Some authors suggest that the effectiveness of oral preventatives is reduced in patients with CA (84). In the same line, the COMPEL study demonstrated that the effect of onabotulinumtoxinA on the reduction of headache days was lower in patients with CA (12). However, the effect of onabotulinumtoxinA on other efficacy measures was similar in patients with and without CA, and the improvement of the MIDAS scale was significantly higher in the former (12). Data available to date do not allow us to conclude with certainty whether the efficacy of preventive treatment is reduced in patients with CA. More studies are needed to clarify this hypothesis. It is possible that in the future, treatments may be personalized based on clinical variables that include the presence of CA.

Considering that main site of action of monoclonal antibodies targeting the CGRP pathway is outside the central nervous system, it is reasonable to hypothesize that these treatments may not be effective in patients with migraine and CA. However, recent studies in migraine patients treated with erenumab for migraine prevention, showed that the presence of CA does not appear to negatively impact on erenumab effectiveness, and the treatment could even reduce the symptoms of allodynia (85, 86).

## Limitations and Strengths

Some limitations need to be acknowledged in the interpretation of our results. First, this is not a systematic review. We limited our scope to selected articles which we believed could be the most representative ones. Secondly, we relied on PubMed and Cochrane only for our search strategy, which has potentially hampered the scope of our narrative review. Among the strengths of our study, we analyzed an important quantity of articles highlighting the most relevant research on CA and migraine.

## Suggestions for Future Studies on Allodynia

Many questions remain to be clarified about CA and migraine. First, it is very relevant to have a deeper knowledge about the pathophysiology of CA to understand why some patients develop CA and others do not. Second, if the longer duration of migraine indicates that CA is a marker for more severe disease, and patients with CA start to have migraine at younger age (32), it can be speculated that children and adolescents with CA and migraine are more prone to chronification at a later stage of their lives. However, this has not been proved, yet and more studies on CA and migraine are needed to verify such correlation in the younger population. Third, the detection of CA can be done easily in clinical practice and therefore the use of ASC-12 should be promoted. Furthermore, the presence of CA may have implications from the diagnostic point of view due to its association with other comorbidities such as depression or medication overuse. It is not known whether treatment of risk factors reduces the risk of CA. On the other hand, it is also important to define the best strategy to effectively treat acute migraine attacks in patients with CA. Finally, if the risk of migraine progression increases in patients with CA, perhaps the use of preventive drugs should be promoted in patients with this symptom.

## Conclusions

CA is common in patients with migraine, reflects the presence of central sensitization, and is associated with migraine progression. The mechanisms underlying CA in migraine are not completely clarified but include a sensitization phenomenon at different levels of the trigemino-talamo-cortical nociceptive pathway and dysfunction of brainstem and cortical areas that modulate thalamocortical inputs. The gold standard for the assessment of CA is QST, but the validated ASC-12 questionnaire is preferred in clinical setting and epidemiological research. CA may increase the probability of a deficient response to acute treatments, but the influence of CA on the effectiveness of preventive medication is uncertain. Clinicians should consider that patients with severe or frequent attacks, who overuse analgesics or with depression, are more likely to develop CA. Addressing these risk factors, may reduce the likelihood of having CA and prevent migraine chronification.

Future studies are necessary to better understand the pathophysiology of CA and to determine how to treat patients with migraine and CA and thus prevent disease progression.

## Author Contributions

AM-O: literature review, data processing and analyses, results interpretation, language editing, review and drafting of the first manuscript, and results interpretation. SQ, NM, AL-B, VG, and MV-P: literature review, data processing and analyses, and results interpretation. RB and SS-L: critical revision of the manuscript. PI: conception and article design, literature review, results interpretation, and critical revision of the manuscript. All authors contributed to the article and approved the submitted version.

## Conflict of Interest

The authors declare that the research was conducted in the absence of any commercial or financial relationships that could be construed as a potential conflict of interest.

## Publisher's Note

All claims expressed in this article are solely those of the authors and do not necessarily represent those of their affiliated organizations, or those of the publisher, the editors and the reviewers. Any product that may be evaluated in this article, or claim that may be made by its manufacturer, is not guaranteed or endorsed by the publisher.
